# Open Access: Pay or Go Away

**DOI:** 10.5704/MOJ.2311.016

**Published:** 2023-11

**Authors:** K Moustafa

**Affiliations:** The Arabic Science Archive (ArabiXiv), Paris, France

Dear editors,

The publishing industry has always been a business, but in recent years, it has witnessed an increasing materialistic trend. This is evident in the way that many publishers are now charging authors with Article Processing Charges (APCs) to publish their work in open access journals that have grown exponentially, leading to both positive and negative impacts on the academic publishing industry.

On the positive side, open access publishing helps make scientific knowledge more accessible, allowing a wider audience to benefit from it by eliminating paywalls and enabling readers to access scholarly articles without restrictions. However, the rise of author-paid open access journals has turned scientific publishing into a profit-driven business^1^ where some journals may prioritise quantity over quality, leading to a decline in the overall standard of published manuscripts^2,3^. The shift of publishing costs onto the authors, rather than the readers, goes against traditional principles of publishing and business^4^, creating financial burdens, particularly for authors in less affluent regions. In the print age, the burden of accessibility was on readers, while in the open access era, it has shifted into an affordability burden on authors.

The claim of free access that open access publishers often boast can lead to misunderstandings among readers, who may assume that the publication process is cost-free, and publishers are generously providing them with such a service. This misconception undermines the understanding of the financial complexities involved in academic publishing and may contribute to a lack of appreciation for the efforts and resources invested by authors.

Another significant challenge in the open access landscape is the emergence of so-called “predatory publishers”. The dichotomy between "predatory publishers" and “non-predatory” counterparts has been a subject of controversial debates over the past years, often overlooking the root causes of the problem. When authors are under permanent pressure to publish more, and when publishers request them to pay high publishing fees, it is not surprising that so-called “predatory publishing” appears to take advantage of the situation with deceptive practices. Predatory publishing cannot be addressed properly without tackling the root causes of the issue, namely the pressure on authors and the associated APCs required for publication.

Initiatives like Plan S in Europe and similar efforts elsewhere, while aiming to promote open access, have incentivised publishers to increase the number of open access journals and establish exclusive open access agreements with institutions, often involving high open access fees. While some journals and publishers claim to waive publishing charges, the reality often reveals a different picture, and waivers are rarely granted. The rubbish 'publish or perish' mantra in the print age is now transforming into a harsher mantra in the open access era that what we can call “pay or go away”, where authors who cannot afford APCs can simply be excluded from the publishing realm.

To address these issues, reforms are necessary to ensure inclusiveness, affordability, and equity, particularly by supporting community-led and non-profit open access initiatives that prioritise accessibility and quality over profit. Finding a good balance between accessibility, affordability, and quality is crucial. The democratisation of knowledge should prioritise empowering a publishing environment where the pursuit of knowledge takes precedence over profit-driven motives. Access to scientific knowledge should be free for authors and readers.
